# Dorsal cervical approach for recurrent intradural anaplastic ependymoma

**DOI:** 10.3171/2023.6.FOCVID2396

**Published:** 2023-10-01

**Authors:** Maxwell T. Laws, Martin Arhin, Leonel Ampie, Prashant Chittiboina

**Affiliations:** 1 Department of Surgical Neurology,; 4 Neurosurgery Unit for Pituitary and Inheritable Diseases, National Institute of Neurological Disorders and Stroke, National Institutes of Health, Bethesda, Maryland;; 2 Department of Neurological Surgery, University of Virginia Health System, Charlottesville, Virginia; and; 3 University of North Carolina School of Medicine, Chapel Hill, North Carolina

**Keywords:** cervical spine, intradural, intramedullary, MYCN, anaplastic ependymoma

## Abstract

The video demonstrates an operative approach to a recurrent cervical anaplastic ependymoma. MYCN-amplified anaplastic ependymomas are locally aggressive, recurrent, and have a high risk of iatrogenic injury. In this case, the patient presented with local, aggressive tumor expansion, arachnoid adhesions, and pial invasion ventral to the spinal cord. Subcapsular decompression minimized cord retraction from a dorsal approach. Removal of the tumor capsule was guided by bipolar stimulation paired with neuromonitoring. Local gross-total resection was achieved, and the patient had a postoperative improvement in his neurological deficits and myelopathy.

## Transcript

Within this video we will describe our operative approach for a challenging recurrent cervical intradural neoplasm.

### 0:27 Patient History.

This is a 34-year-old gentleman with a past medical history for a WHO grade 3 ependymoma which was diagnosed in 2012. Initial resection was done in May 2012 with a C1–4 laminectomy. He developed iatrogenic cervical kyphosis, and subsequently underwent an anterior and posterior cervical fusion in 2013.^1^ Throughout this time, he received adjuvant chemotherapy and radiation. In 2016, he had progression of his cervical lesions and in 2017 he was started on nivolumab immunotherapy.^2^ He presented to us in February 2018, with worsening left-sided weakness, gait instability, sensory loss on the left, and both bladder and bowel incontinence.

### 1:17 Neurological Examination.

On neurological exam, the patient had weakness in his left upper extremity, most prominent in the C7–T1 myotomes. He was also markedly myelopathic and hyperreflexic, along with diminished sensation throughout the left upper and left lower extremities.

### 1:37 Description of Preoperative Imaging Findings.

MRI of the cervical and thoracic spine showed multiple enhancing intradural nodular masses, the most prominent lesion was at the C3–5 level on the left with significant edema and mass effect.

### 1:56 Operative Planning.

The patient was consented for a redo posterior cervical approach for tumor resection with the intent of achieving local tumor control and improvement of his neurological deficits caused by the tumor mass effect. The patient’s head was secured in a Mayfield clamp with three-point fixation. Neuromonitoring leads to record SSEPs and MEPs were placed and a localizing x-ray was performed.^3,4^ A surgical incision was made at the prior surgical site and Bovie monocautery was used to dissect subcutaneous layers and expose the prior laminectomy cavity. The wound was filled with irrigation and intraoperative ultrasonography was used to identify the rostral and caudal extent of the tumor.

2:43 Surgical Approach. The dura was identified and opened sharply with an 11 blade and a grooved dental instrument. The dura was undermined and adhesions to the arachnoid were released. Intraoperative ultrasound demonstrated a CSF cleft between the arachnoid and dorsal aspect of the spinal cord. The arachnoid was separated off the tumor circumferentially using microdissecting bipolars set at a low heat. Ligaclips were used to adhere the dura to the arachnoid. A pial plane was established between the tumor and cord using microsurgical technique. At this point we could clearly see the dorsal border of the tumor and spinal cord. The tumor exhibited an insinuating growth pattern, with dentate ligament noted to bisect the tumor at the C4 and C5 level. These ligaments were incised to expose the ventral aspect of the tumor which had more extensive pial invasion and adhesions. Bipolar stimulation was used to direct the resection and navigate around ventral nerve rootlets.

### 4:05 Subcapsular Decompression.

Since this was a case of a recurrent ependymoma and there were significant adhesions present, the decision was made to perform a subcapsular decompression in order to minimize cord retraction.^5,6^ We coagulated and sharply incised the tumor capsule and commenced internal debulking using Rhoton microdissectors, bipolar cautery, and ultrasonic aspiration. We cauterized the dura which was associated with the ventral tumor capsule. We performed a subcapsular dissection circumferentially along our established pial and capsular planes. A ventral nodule was identified adjacent to the nerve rootlets at C4; this was carefully removed under the guidance of bipolar stimulation. The tumor cavity was inspected and there was no visible tumor remaining. Having achieved local gross-total resection, hemostasis was obtained and irrigation performed. The dura was closed in a watertight manner using running 4-0 Nurolon, and standard multilayer closure was performed using Vicryl interrupted sutures, followed by a running 3-0 nylon suture for skin.

### 5:39 Review of Postoperative Images.

Postoperative MRI showed successful local gross-total resection and resolution of the mass effect at C3–5.

### 5:49 Postoperative Course.

The patient was discharged uneventfully on postop day 4. He noted improvement in both his strength and sensation on his left side. At 3-month follow-up, he had improvement in the strength in his distal left arm and had general improvement in his sensation and hyperreflexia. Molecular testing including an Oncomine assay confirmed MYCN-amplified anaplastic ependymoma portending a poor prognosis.^7^ In June 2018, he demonstrated interval growth of a dorsal T9–10 lesion with worsening lower-extremity weakness, along with bowel and bladder dysfunction and underwent a T9–10 laminectomy and tumor resection. Two months later, he noted worsening left upper and lower extremity weakness and was found to have interval expansion of a right craniocervical mass, which was resected in August 2018 with a far-lateral approach. His lesions continued to progress, and he trialed oral vorinostat and subsequently failed. He opted for hospice care and passed in June 2019.

## Acknowledgments

This work was supported financially by the Intramural Research Program of the National Institute of Neurological Disorders and Stroke and the National Cancer Institute in Bethesda, Maryland.

## Disclaimer

The authors have no personal, financial, or institutional interest in any of the drugs, materials, or devices described in this article. The views expressed are solely those of the authors and do not reflect the official policy or position of the National Institutes of Health, the US Government, or the University of North Carolina School of Medicine.

## Disclosures

The authors report no conflict of interest concerning the materials or methods used in this study or the findings specified in this publication.

## Author Contributions

Primary surgeon: Chittiboina. Assistant surgeon: Ampie. Editing and drafting the video and abstract: all authors. Critically revising the work: all authors. Reviewed submitted version of the work: all authors. Approved the final version of the work on behalf of all authors: Laws. Supervision: Laws, Chittiboina.

## Supplemental Information

### Patient Informed Consent

The necessary patient informed consent was obtained in this study.
